# We Made Better Dentists Then Than Now

**Published:** 1904-01-15

**Authors:** D. R. Stubblefield

**Affiliations:** Nashville, Tenn


### "WE MADE BETTER DENTISTS THEN THAN NOW."

BY D. R. STUBBLEFIELD, M.D., D.D.S., NASHVILLE, TENN,

Read before the Academy of Stomatology, Philadelphia, March, 1903

This startling quotation is from one of the best men in American dentistry, For more than twenty years he has been admired as a man, a teacher, and a dentist, one whose life traditions gave warrant of excellent ability, whose aspirations considered the highest attainment only as worthy, and whose associations argued conclusively that his profession might look upon him with confidence and pride.

On the above occasion it had been claimed that the addition of an other college year gave a much needed opportunity to add important phases of modern scientific knowledge to the college curriculum. The enthusiast who proposed had been followed by others, until the roseate dawn of a newer and better era in dentistry was flung upon the imaginary canvas of our professional East and the kindling enthusiasm of the moment was beginning to spread like any other conflagration. Then it was that this certain man spoke, among other things, the sentiment which has been considered worthy of your interest to-night. He is no sentimentalist. He said what he did from no desire to detract from the dignity and worth from what is done to-day, from the standpoints of intrinsic scientific value, but he gave the sober judgment of a thoughtful mind. For more than twenty-five years he has satisfactorily filled the the demands of an intelligent, progressive teacher, and from this eminence, this coign of vantage, having surveyed the whole field, he uttered the conviction that *better dentists were made in the past than to-day.*

Is it true? Is it only partly true? Or is it without foundation in fact? Do you, young men, believe that those who preceded you came upon the field of professional life better equipped, better able to discharge its duties, than you who may rightly claim to be the exponents of a later, more progressive *regime*? In the light of this day of boasted scientific inquiry and investigation it is a bold, if not a rash man who can utter such a sentiment. But, again, I declare that the man who said it is worthy of your confidence; in truth, that man holds your confidence to-day, and is entitled to that distinction from every single step between sentiment and sense.

Before others may have the floor, what can be said about this proposition that "We made better dentists then than now?" If he is correct, a clear understanding of his reasons for belief may be of much value in settling the problem of the four-year law. For the purpose of this discussion let me assume the responsibility of this utterance and offer myself awhile, at least, as the victim upon the altar of investigation. I say this in sound mind and disposing memory, with a full consciousness of the enormity of the offense and with a just apprehension of the dire fate that may await me. The sergeant-at;arms has been duly instructed to lock and bar the doors from the outside, and no men of you can escape hearing this suicidal confession, even if it causes the marrow of your bones to freeze and the hair of your flesh to stand up with chilling horror. I am determined to do it, even if I, reportorily, scatter my brains and spatter my heart's hot blood upon your innocent heads. I, who am about to die, salute you!

In all seriousness, hear his cause. There is some foundation in fact to the claim that better dentists were made in other years than right now. From the standpoint of an interested observer for almost a quarter of a century, I am ready to believe that the modern introduction of so much or so many varied branches of science has tended to obscure the clearness of sight and definiteness of grasp upon the great fundamental idea of all dental instruction. It may be asked, Do you mean to antagonize progress and modern scientific development? Do you attempt to deny that the wealth of scientific contribution to the course of instruction has enriched the minds of those upon whom it has been lavished? If not, how can you hope to maintain such a conclusion that is all but iconoclastic in its effect upon that awe-inspiring pile of knowledge, otherwise known as the up-to-date dental college curriculum. I can not deny its richness, its wealth of scientific attainment, and for that reason I do not make my vicarious substitution unconditional. But I do claim that such profundity of knowledge, such wealth of science, do not necessarily give the coming dentist a commensurate degree of excellence in rendering practical the golden idea of greatest fundamental importance and make him of necessity the ideal exponent of its greatest beneficence to mankind. To my mind, the cart is before the horse. If such extensive acquisition of scientific knowledge could be made to exert its enriching power before the student has set himself to acquire that perfect technic which alone can make him the ideal practitioner, it would be better. Then the proper relation between scientific learning and practical performance would be better established. Then the student would, indeed, be more able to see broadly and decide maturely the application of means to ends and the scientific reason for them.

If it be true that the introduction of even valuable material may obscure the vision or the practical simplicity of the end aimed at, then I hold that the instruction of other years which was not interfered with by what may be called an embarrassment of riches was able to produce a stronger, clearer practitioner than the gradutae of to-day can possibly claim to be at the outset of his career. A practitioner of any calling is essentially a *doer*. It is, *therefore*, unthinkable that any exponent of a practical utility can be unable to do, to make, to accomplish. Just so long as the college course held as its highest, if not its only, ideal the attainment of power to do, it was naturally productive of a better development in that direction. At that time its appropriation of really valuable knowledge from cognate sciences was dominated by the ruling idea of perfect practicality. If I may use a homely figure, just so long as these scientific outsiders were used as condiments or flavoring extracts, they contributed to the general good effect of the whole, but when they became, or were dangerously near becoming, a preponderating component, they spoiled the whole pudding. If I may be pardoned, I believe I can make my meaning clearer by a personal, illustration. My teacher of physiology was a most erudite man, so full of physiology and other sciences that to save his life he could not restrain himself to the bare, simple principles of physiology which my crude mind could hold and digest. From his standpoint he adequately covered the field and filled the measure of demand so full it overflowed at every side. But one poor student found in a modest quiz-master, who only knew the headlines, the landmarks, the great and simple facts that beaconed the land of Physiology, his only salvation. The quiz-master knew only just enough to get to the end and go back again, refreshing the mind with the reiterated principles until all the salient facts of that most beautiful branch remain in my mind to this day like a twice-told tale. There you have it. Just so long as the teachers of dentistry had clear minds to teach the essential ideal of making a dental *practitioner*, just that long were they able to impart definite instruction which comparatively untrained minds could and did grasp and digest.

I say it is no reasonable claim that the comparatively simple course of the earlier years was more appropriate to the simple and untrained minds which presented themselves at college, and, unless it can be clearly proved that the cultivation of the material now in the classes has proportionately improved, that the more complex courses, filled to bursting with excellent things, are still beyond the mental assimilation of the average student. There has been much improvement educationally, but not enough yet. The classes have grown younger and therefore more impressionable, which is a distinct gain, but receptivity of mind, engendered by training, must be attained before the best results can possibly be obtained.

The mental attitude of the student has changed in late years. This is hard to prove, for so many personal exceptions suggest themselves that the mind can not accept the principle broadly. In the earlier years, when there existed in the mind of the world the idea that dentistry was not quite as respectable as some other callings, the ranks of dentistry were recruited by those whose minds were more strongly directed than those of to-day. They became dentists because they wanted to be dentists more than anything else in the world. To-day we have many who come into college because some single exponent known to them is supposed to be prospering greatly and yet can claim no superiority to them, who may be at the time "working themselves to death for nothing."

The mental attitude of the self-elected, who listened to the outcry of nature, is infiinitely superior to those even better educated and more pliable of mind who are actuated by a motive no higher than a commercial basis. If this is true, it can not be entirely unreasonable to claim that the average professional practitioner was better from that other *regime* than the average graduate of to-day who may be admitted to be a much fuller man. We cast no reflections upon the intrinsic worth of all scientific knowledge. We would not if we could say or do anything to minimize the just estimate placed upon the wonderful reaches of modern science. We would not have it thought that we would undervalue the great benefit that dentists may obtain from such broadest cultivation. The intelligent practitioner of dentistry can not be too broad-minded nor too far-seeing to meet and solve the protean problems which daily confront him. The question is not how much has been supplied to him that was valuable, but rather how much knowledge has become the most wisdom to him? Knowledge is power, but it is only stored ammunition, passive potentiality; but wisdom is possibilities in action, mighty in effect for good or for evil. We must not measure nourishment by ingestion, but by digestion, and we would rest our claim, if we have any at all, for the correctness of my text upon the single idea that the average student in other days more nearly digested his course than is the case to-day.

As a practical teacher I have hailed each addition to the curriculum as another opportunity of value to the student. I have not always, however, felt certain that the practical value of the course would be necessarily increased by such valuable extension. The value is there—that I did not question—but I could not feel certain that its full cultural possibilities would become tangible. Personally, I have been a stickler for broader, better education as the best foundation upon which to erect the superstructure of any professional equipment. Good education, however, is not measured by number, or length, or complexity of the courses, but by results, by increased power to think, by the cultivation of the powers of the mind, whether that has been accomplished by college methods and courses or not. The ability to grasp easily what is presented, to understand the teacher readily, not only as to what is said but what is meant, may be largely a matter of personality, but leading out the mental powers by education more certainly achieves that than anything else. Such skilled intelligence is what I have longed for and even hoped for in our classes. But now that condition of mental parts is most usually if not better obtained from collegiate instruction, and for that reason I have exulted when I saw that the trend of the action of the National Assoication of Dental Faculties was toward more definite educational instruction. Let me again use a homely figure. We may have the best seed as to quality, variety and adaptability, and we may have in addition soil that promises the best results, but no farmer would hope to reap a harvest if he did not carefully prepare the ground. On the other hand, with thoroughly prepared earth fair results may be obtained even if it must be confessed that the farmer himself was not the wisest. The point I wish to make is that if we, the profession, can help the schools to so present the various subjects of the course, with especial relation to the preparation of the average student to understand the value of each part, this four-year law will certainly bring a great uplift to our professional future. Let us emphasize the importance of maturely weighing each branch and then intelligently placing it in the logical sequence of its relative importance to our scheme, and we shall greatly assist the cause of professional education. If we examine critically we can show, I dare say, that most of our courses have become heterogeneous aggregations of material good enough in itself. The fault is rather in the lack of system, lack of logical sequence, rather than lack of satisfactory, substance. Education-building is not altogether dissimilar to house-building so far as the methodical sequence is concerned. It would be the grossest folly to attempt to put a part of the roof in the foundation, or, as I greatly fear we have been compelled to attempt to do, try to build the professional roof without any foundation under it. I would not have you suppose I undervalue the actual improvement of the course in the twenty-four years of my effort to teach, but in this heart-to-heart talk with you I want you to know that I have sometimes feared we were not getting the best results possible out of the opportunities we did have. This is said with special reference to those additions from the correlative sciences. With reference to those developments along the line of technic or the manual training in the art of dentistry, there can be no discussion. The inner consciousness of every single older dentist tells him that all of that most excellent practical cultivation would have been an inestimable boon to his young professional years. But I speak of those branches which, from a broad scientific standpoint, lend a distinct cultural insight into our special field of interest. If we could get all the maturing of mind and the training of intelligence desirable from their broadening influence, then all could build confidently; then we should reap the richest harvests or feel ourselves to blame for the deficiency.

This is the message I bring, a plea for a more logical rearrangement of the present curriculum, and thereby obtain a better use of all the excellent materials collated there, to the end that those entering our profession may, in fact as well as semblance, be *educated men.* Time is a very important factor in material digestion, and should not be deemed less so in the question of perfect educational assimilation. The failure to obtain satisfactory results is generally due to more than one cause. Starvation is most horrible when it is attended by the tantalizing association of copious ingestion of food, but it is possible. Therefore, the addition of another session gives the assurance of a better opportunity than ever before to extract the full measure of professional nutrition from the courses in our colleges.

### DISCUSSION.

Dr. E. C. Kirk: I do not believe that the gentlemen who uttered the statement quoted as the title of the essay meant what that title would be taken to mean read as a bald statement. It must need qualification from the fact that the essayist says it was a statement made by one of the best men in dentistry. It seems impossible that in our growth of knowledge of detail which makes up our educational system we have taken a backward step. If the statement means that we actually did make better dentists then than now, I frankly state that I do not believe it; because within my recollection it was quite customary to talk about such things as "laudable pus." We know now that pus is not laudable. We knew very little about the conservative treatment of the dental pulp. We have gone the whole gamut, and are beginning to view the operation of pulp-capping somewhat askance. We knew little about the etiology of dental caries, though men would attempt to explain the tendency of teeth to decay on the proximal surface upon the theory of lateral pressure. It may seem insignificant that a wrong conception of theory should have any particular bearing on our work, but as an outgrowth of that conception we had methods for the absolute mutilation of the approximal surfaces, because men beleived in the production of caries by proximal contact. It took some years of scientific education to get rid of that heresy, and upon this newer education we have based a rational mode of practice. The men who know these things are better dentists than those of years ago who did not know them. In regard to manipulative skill, they had some wonderful operators then. I was talking with a man from Kansas City on this topic, and he said that twenty-five years ago he was the only dentist in Kansas City capable of putting in a decent contour cohesive-gold filling. To-day there is scarcely a village throughout this land where we will not find a man capable of doing this work. I think that twenty-five years ago there were more men especially called to practice dentistry in the sense that they had natural ability and enthusiasm for their work. I read recently in an old copy of the New York *Dental Recorder* an adverstisement of the old Baltimore College of Dental Surgery. The winter courses were from November to February, and the men attending the medical course were permitted to come up for the degree at the end of one winter's course. Dr. Stubblefield said they assimilated the courses better then than now. Naturally, for they did not have much to assimilate. The data of dentistry had not grown then to present proportions. The men who make this comparison to the detriment of modern dental conditions seem to overlook the fact that the data has accumulated to such an extent that it is beyond the ability of the ordinary mind to grasp it at once.

Dr. James Truman: It is a habit, I believe, with all the older men to say that the former days were better than these. The older days, in some respects, were better than these, and the present is infinitely better in other respects, than the older days. We must make a distinction. When we go back to the very beginning of the dental profession, the origin and formation of college life, we realize how limited was the instruction. The man who wanted to study anatomy, for instance, received but a modicum of the science. Chemistry was not, physiology was not, and, may I say it? mechanical dentistry was not. This was because the men who came into dentistry along in the fifties were men who studied under other dentists, who received their mechanical education under the fathers of the profession. and coming into college they cared nothing for the mechanical and a great deal for the operative instruction. The mechanical part was neglected, and up to a period in the seventies there was practically no mechanical dentistry taught at any of the colleges that I was acquainted with. Then came a change, and if there has been any change that has been to the advantage of the dental profession, it has been that the technic of mechanical dentistry has been more thoroughly elucidated during the last twenty-five years. In my opinion, in proportion as that branch has advanced, operative technic has lost ground. This is probably because we have undertaken to do too much in this present day. I am in sympathy with the broader education of the present time, but I am sure that the men do not digest what they receive. There is but a partial education all the way through. If you want a man to know anything at all you must drill it into him, and that can not be done in a few days or a few weeks. He must have time to digest that which he has taken into his mental organism. We have not had sufficient time for that, and this is the reason why we are not making better practical dentists to-day then were made when I was young in dentistry.

The operative branch to-day is not equal to what it was forty years ago. When I say that I mean that we know how to fill teeth fifty years ago. We did not have all the the appliances that we have at the present time, and it required more skill with the few that we had at that period. The past was the foundation upon which all the present is built. We must endeavor to arrange the curriculum that the individual will take that which seems best adapted to his work and endeavor to do it thoroughly. I do not want to be misundertsood here. I am just as much in favor of that broader education as my friend Dr. Stubblefield, or as Dr. Kirk, but I want every individual to be as thorough as possible in what he acquires.

It has been said that men were older when they came into the profession a few years ago. That is true. They were older because they had been with their preceptors. We find to-day a better educated class of men entering the profession, but they enter without any idea of their fitness to become dentists. I regret we have such a class of students to deal with. Twenty-five or more years ago men knew why they wanted to become dentists; now it is more often what the parents or guardians desire.

DR. S. H. GUILFORD: It is very difficult to decide a question of this kind on account of the many problems presenting. There is something to be said on all sides. As has been said, men who studied dentistry twenty years ago were men who had some decided taste for this sort of work. This was shown in the boy who would take clocks apart or meddle with locks or water-wheels. In my own case I was led into dentistry simply because I was constantly tinkering at something. At that time, too, the boy entering dentistry was put on a system of probation to ascertain whether he was fitted for dental work. If he was not, he was dropped. To-day, young men enter into dentistry for the reason that they think they can make more money and make it more easily than in some other way. Looking at the matter in that light, I believe more good dentists, proportionately, were turned out forty years ago than to-day.

In regard to the making of dentists, colleges did not make the dentists; the dentists made themselves. When I went to college we had a good course in anatomy, but of the other branches we did not have very much that we could be proud of. We secured, however, a little foundation upon which we could build. If I know anything of dentistry to-day, I have learned it nearly all since I left college. It is not necessary that our students assimilate all of the curriculum; if we take them over the curriculum and get them into the position where they can learn after leaving college, we are doing well with them. A man must have ability and knowledge, and if he has the ability he will work, and there is no reason why he should not be a success. I wish there was some way to-day of finding out when a student enters college whether he has natural qualifications for the practice of dentistry. Any profession is better when made up of men especially fitted for the work. I do not think the number of branches in the curriculum needs to be increased, but more thorough drilling in the present branches is needed.

Dr. E. T. Darby: I was present in Chicago at the Institute of Pedagogics, and heard the statement made which has been the text of the evening. It was made when the subject for discussion was "The Fourth Year in Dental Schools." I also heard the gentleman who made the statement modify it at the close of his remarks by saying that we made better practical dentists twenty years ago than we make to-day. The statement was made with the idea of emphasizing the importance of giving the student in the fourth year a great deal of practical work. I heard this same man say at that time that no one was more in favor of broad culture, of thoroughness in training, than himself, and that he believed in giving the student everything that it was possible to give him in the way of culture commensurate with making a good, practical dentist of him. I do not believe that that man believed in giving the student less, but in giving him more practical work.

It is by doing over and over and over again those things that we do with our fingers that we become dexterous in their doing. We have crowded into our three years so much that is valuable, so much that students ought to know, that we have sacrificed to a large extent the time that ought to be devoted to practice. For that reason I believe that that statement was true, that when twenty years ago we had very much less in our curriculum than we have now. and expected very much less of our students than we do to-day, and when they devoted so much more time to work in the laboratory or in the clinic-room, they were better practical men than the men turned out to-day.

For this reason I shall hail with delight the addition of the fourth year to our course. I would not take anything from our present college course, but I would make the men good, practical dentists before we send them out. If the men who have a fitness for dentistry would enter our schools, we would have no difficulty in turning out good dentists.

We will make better dentists with the four-years' course simply because we will give the students more practice in addition to the better scientific foundation.

Dr. M. H. Cryer: I believe that to-day I am a better dentist than yesterday, and that this year we will turn out better dentists than we did last year, and that last year we turned out better men than we did the year before. We treat teeth better than we did formerly. We take better care of the mouth and the system than we ever did, and I believe that we are advancing all the time. It is not the time to look at what is past. I believe that as we advance and add to our knowledge the things that are necessary we will make better dentists than we did in the past.—*International.*

